# Mapping public health research across the National Institute for Health Research 2006–2013

**DOI:** 10.1186/s12889-016-3521-z

**Published:** 2016-08-31

**Authors:** Eleanor Woodford Guegan, Hannah Dorling, Liz Ollerhead, Matt Westmore

**Affiliations:** NIHR Evaluation Trials and Studies Coordinating Centre (NETSCC), University of Southampton, Alpha House, Enterprise Road, Southampton, SO16 7NS UK

**Keywords:** Public health, Research, Funding, Programme evaluation, Taxonomy

## Abstract

**Background:**

Public health research is an important component of United Kingdom (UK) health research and strategic analysis of its breadth and balance is key to ensure value. The National Institute for Health Research (NIHR) is one of the main funders of health research in the UK and includes many research programmes and schools. This study reports on public health research funded by the NIHR between April 2006 and March 2013.

**Methods:**

The NIHR research programmes and schools were asked for information about all research funded during the study period. Firstly, projects were classified as a public health research project according to inclusion and exclusion criteria. The public health research projects were further categorised according to the Public Health Outcomes Framework and the National Institute for Health and Care Excellence taxonomy.

**Results:**

Approximately 3000 research projects were funded by the NIHR, of which about 900 were relevant to public health. This represents approximately one-third of the research portfolio. All NIHR research funding programmes and schools funded research related to public health. The most prevalent domain of the Public Health Outcomes Framework was ‘healthcare public health and preventing premature mortality’ and there were a large number of health planning and self-management projects. One-quarter of projects were concerned with mental health and behavioural conditions.

**Conclusions:**

The NIHR is a significant funder of research relevant to public health. This analysis offers a snapshot of the breadth and balance of NIHR research, which forms a basis for discussion. This is important for the NIHR and other research funders as it shows areas that are better represented and opportunities to fill important gaps. Appropriate research priority setting is an integral part of a needs-led research agenda and adds value to research.

**Electronic supplementary material:**

The online version of this article (doi:10.1186/s12889-016-3521-z) contains supplementary material, which is available to authorized users.

## Background

It is vital that appropriate public health research is funded to improve population health. Strategic analysis of the breadth and balance of public health research is key to ensure value, particularly as it represents a significant part of UK health research. Analysis of funded research is essential when decisions are made in relation to what further research is needed. Chalmers et al. explain that such assessment will “identify what should be replicated, avoid unnecessary duplication and result in research that addresses deficiencies in previous work” [1].

Analyses have been performed by several groups in the UK with regards to public health research. The UK Clinical Research Collaboration (UKCRC) published a report in 2012 about health research funded in 2009–2010 by the 12 main public and charitable funders of health research in the UK [2]. They used the Health Research Classification System (HRCS) to classify almost 12,000 peer reviewed awards. Information was included from charities, health departments and research councils. Three-fifths of the funded research focussed on the basic understanding of health and disease, whilst only 4 % of research was preventative in nature; research into the primary prevention of disease or conditions, or promotion of wellbeing. The Wellcome Trust published a portfolio review in 2013 of their Population and Public Health projects funded 1990–2011 [3]. They reported that funding for this area of research accounted for 9 % of their total research portfolio, with almost half of these studies being classified as within the sphere of ‘infection’ using the UKCRC Health Research Classification System.

There have also been several important European initiatives in this area. Strengthening Public Health Research in Europe (SPHERE) was a 3 year project (2005–2007) funded by the European Commission [4]. Membership was a consortium of European partners, of 19 partners from 12 European countries, including the UK [5]. A review of bibliometric research across several public health areas identified that approximately one-third of world outputs in public health research were contributed by Europe [6]. The UK was found to have the highest numerical output of public health research within Europe [6]. Using a broad definition of public health, ministries were asked how public health research priorities were set, how funding was assigned and results disseminated. Ministries cited from 3 to 30 research themes, in terms of areas of research (e.g. including research on prevention and health promotion), diseases (e.g. cancer) and determinants of health (e.g. social inequalities).

The Public Health Innovation and Research in Europe (PHIRE) project was a 30-month project performed between September 2010 and February 2013 [7]. It was part funded by the EU Health Programme and coordinated by the European Public Health Association [7]. It identified 75 public health research programmes and calls across 16 countries that opened in 2010 [7]. Analysis of the UK contribution to PHIRE identified that 15 programmes and calls for public health research were made in 2010 [6]. The main funders in the UK were the National Institute for Health Research (NIHR) and the Medical Research Council (MRC) and research was funded across a range of fields, including health promotion, health services epidemiology, surveillance, management and wider determinants [6].

Such a mapping exercise has also been performed in other European countries, most notably in Scandinavia. An inventory of Swedish public health research funded between 2000 and 2003 was published in 2005 [8]. Over three-quarters of projects investigated aetiology. A similar descriptive analysis of public health research in Denmark identified that 209 research projects had been funded in a 10 year period from 1995 to 2005, with the most frequent category being in health promotion [9].

The NIHR is one of the foremost funders of health research in the UK [10]. This paper reports an analysis of the NIHR funding of public health research over a 7 year period.

The NIHR was founded in 2006, following publication of the Strategy Document “Best Research for Best Health” [11]. The NIHR’s role is “to provide a health research system in which the NHS supports outstanding individuals working in world-class facilities, conducting leading-edge research focused on the needs of patients and the public” [12]. It is a large, distributed organisation. The various research programmes and schools of the NIHR are summarised below in Table 1.

**Table 1:** Research Programmes and Schools of the NIHR

Programme/School	Focus
Efficacy and Mechanism Evaluation (EME)	Bridges the gap between preclinical studies and evidence of clinical efficacy. The aim is to secure the progress of new technologies and interventions through their early clinical trials and onto larger, later clinical trials.
Health Services and Delivery Research (HS&DR)	Research to produce rigorous and relevant evidence on the quality, access and organisation of health services, including costs and outcomes.
Health Technology Assessment (HTA)	Research about the effectiveness, costs and broader impact of healthcare treatments and tests for those who plan, provide or receive care in the NHS.
Invention for Innovation (i4i)	A translational funding scheme to advance healthcare technologies and interventions for increased patient benefit in areas of existing or emerging clinical need. New and Emerging Applications of Technology (NEAT) and Health Technology Devices (HTD) are historic schemes, now managed under the umbrella of i4i.
Programme Grants for Applied Research (PGfAR)	Produce independent research findings that will have practical application for the benefit of patients and the NHS in the relatively near future, through promotion of health, prevention of ill health, and optimal disease management (including safety and quality). A particular emphasis on conditions causing significant disease burden.
Programme Development Grant (PDG)	A complementary scheme to PGfAR. This allows investigators to undertake preparatory research that will position them to submit a competitive Programme Grant application.
Public Health Research (PHR)	Research that evaluates public health interventions, providing new knowledge on the benefits, costs, acceptability and wider impacts of interventions outside the NHS, and intended to improve the health of the public and reduce inequalities in health.
Research for Patient Benefit (RfPB)	Inspired by patients and practice to generate high quality research for the benefit of users of the NHS in England. Its main purpose is to realise, through evidence, the huge potential for improving, expanding and strengthening the way that healthcare is delivered for patients, the public and the NHS.
Research for Innovation Speculation and Creativity (RISC)	Provided research of direct benefit to users of the National Health Service (NHS) in England. The RISC programme is a historic scheme that was for potentially paradigm-changing projects in Health Services and Public Health Research.
Schools for Primary Care, Social Care and Public Health Research	The three Schools each aim to: increase and develop the evidence base for practice in the primary care, adult social care, and public health sectors respectively, build research capacity, improve research awareness and create a ‘critical mass’ of research expertise and funding through coordinated and collaborative working across the country.
Systematic Reviews (SR) Programme	Consists of a number of initiatives, including the Cochrane Review Groups and the UK Cochrane Centre, the Centre for Reviews and Dissemination and the Health Technology Assessment Reviews, which provide high quality research evidence to support decision-making.
Trainees’ Coordinating Centre (TCC)	Makes training awards to researchers whose work focuses on people and patient-based applied health research. This research training is funded in order to build a leading NHS Research Faculty, develop research careers, research leaders and collaborators.

In addition there is collaborative funding with the Policy Research Programme (PRP) which commissions high quality, research-based evidence relevant to the full policy remit of the Department of Health (DH). PRP research is commissioned by open competitive tender within the DH Research Governance Framework (2005).

### Public Health Research within the NIHR

The NIHR funds a range of research programmes and schools that address and evaluate major public health questions including, for example;

- NHS interventions delivered to whole groups with the aim of improving population health (such as immunisation and vaccination).
- NHS services and initiatives targeted at individuals at higher risk of future ill-health to reduce their risk (such as smoking cessation).
- NHS interventions aimed at identifying those with early stage asymptomatic disease where early treatment improves prognosis (such as screening).
- Health interventions outside of the NHS aimed at a group or population level (such as school-based healthy eating programmes or changes to the built environment).
- The organisation and delivery of healthcare with the aim of improving service quality and patient safety (such as access to healthcare).
- Interventions that aim to reduce inequalities in health (such as those that aim to improve working and living conditions).

The PHIRE project reported that the NIHR is one of the two main funders of public health research in the UK [6]. Therefore, it is interesting to analyse its research portfolio in greater depth. There is little information available in the academic literature about the overview of the breadth and scope of public health research from a funder perspective. This study reports on the range of public health research funded by the NIHR from its inception in April 2006 to March 2013. This is of interest to researchers in the public health community because it identifies the place of the NIHR among other funders of public health research in the UK. It gives an indication of the broad range of public health research funded and highlights the number of research programmes which can potentially be applied to for funding. It is also valuable for the NIHR to reflect on the breadth and balance of public health research it funds. This will help to evaluate what progress has been made, and help to inform future strategic discussions about research gaps.

## Methods

**Fig. 1:**
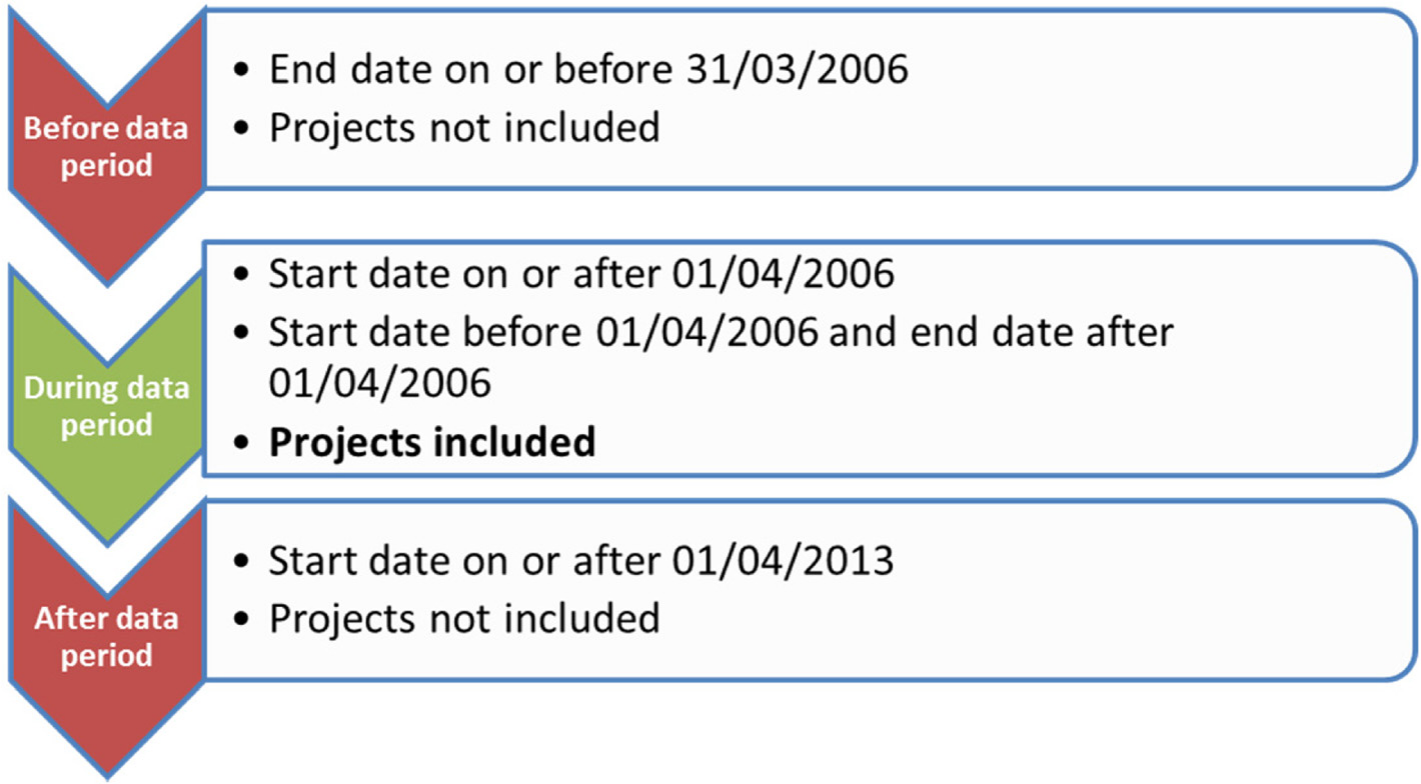
The timeline for inclusion of projects in the study

A portfolio analysis was performed of public health research funded by the NIHR during a 7 year period. The NIHR research programmes and schools were asked to provide information about all the research projects they had funded since the inception of the NIHR on 01/04/2006 to 31/03/2013. The ‘active period’ of a research project was the time between its start date and end date. Figure 1 illustrates the inclusion criteria of projects by start and end date.

The following information was requested for each research project;

- ID reference
- Funding programme
- Name of principal investigator
- Start date
- End date
- Funding awarded

Research projects were categorised according to whether they were classified as public health projects. These projects were then categorised according to public-health specific coding systems.

### Classification as Public Health Research

Public health is a broad concept which comes from a variety of disciplines and can have a range of meanings. Colleagues, including those with a public health background, had varying ideas about what constituted a ‘public health’ research project. Therefore it was necessary to derive inclusion and exclusion criteria (see Additional file 1). It is important to note that these criteria were developed by the authors and do not necessarily represent the views of other public health professionals, researchers or the NIHR organisation. The authors recognise that the development of these criteria is an imperfect science and a pragmatic approach had to be taken.

The title and abstract of each research project was assessed to identify those that met the inclusion criteria for public health. Those projects, which were classified as ‘public health research projects,’ were stored in a Microsoft Excel® version 2010 spreadsheet for further analysis.

### Classification according to the Public Health Outcomes Framework 2013–2016

The next stage in the project was to further categorise these ‘public health research projects’ according to the Public Health Outcomes Framework (PHOF) for England 2013–2016 [13]. This was developed by the Department of Health in 2012 and sets the context for the public health system from the local to national level. This document includes a set of public health indicators which are grouped into four domains, covering the full spectrum of public health. Those projects that were classified as ‘public health research projects’ were classified into one or more of the four domains, and these were coded on the database.

Information on the four domains of the PHOF is shown below in Table 2.

**Table 2:** The domains of the Public Health Outcomes Framework for England, 2013-2016

	Domain 1	Domain 2	Domain 3	Domain 4
Definition of the domain	Improving the wider determinants of health	Health improvement	Health protection	Healthcare public health and preventing premature mortality
Objective of the domain	Improvements against wider factors that affect health and wellbeing, and health inequalities	People are helped to live healthy lifestyles, make healthy choices and reduce health inequalities	The population’s health is protected from major incidents and other threats, while reducing health inequalities	Reduced numbers of people living with preventable ill health and people dying prematurely, while reducing the gap between communities
Examples of types of research projects included	Housing, environment, employment	Health behaviours, such as physical activity or smoking	Patient safety, infection control	Screening programmes, improving services

It was useful to code the data using a further classification scheme, to enable greater data analysis. It is important to highlight that unfortunately there was no coding scheme specific for public health available at the time of the study. Therefore a pragmatic approach had to be taken. Several taxonomies could have been used and the following possibilities were identified;

- Medical Subject Headings (MeSH®) is the National Library of Medicine’s (NLM) controlled vocabulary thesaurus. It is used by NLM for indexing articles from biomedical journals for the MEDLINE/PubMed Database [14].
- The Public Health Language is a taxonomy specific to public health. However, at the time of the study it did not appear to have been updated since 2008 [15].
- The National Institute for Health and Care Excellence (NICE) taxonomy is a subject encoding classification scheme providing a consistent language across NICE to support website information and NICE guidance [16].

The MeSH® system was not used for this project because it was not deemed to be specific enough for public health, and Public Health Language was considered not sufficiently current to be used. Therefore, the authors decided to use the NICE taxonomy as this was current at the time of the study and there are professional links between NICE and the NIHR.

The version of the NICE taxonomy used was prepared on 14 June 2012 and contained over 1700 terms. The taxonomy is multi-hierarchical, which means that a term can appear in more than one place, which is a limitation of using this scheme. One example is the term ‘bowel cancer’ which appears under both ‘cancer by site’ and ‘gastrointestinal diseases’.

Within the taxonomy, ‘public health’ was categorised into;

- health behaviour,
- public health practice,
- and socioeconomic determinants of health.

Fields were included in the Microsoft Excel® database for these categories.

Extra fields were included by the authors to provide richer data about the public health research projects, which were;

- treatments,
- procedures and devices,
- illness or condition,
- and setting.

The titles and abstracts of the public health research projects were reviewed and the appropriate codes added to the database. Tables defining the categories and levels of the codes for each field are included in Additional file 2.

### Quality assurance for classifying projects as ‘public health research projects’

The more difficult part of the three-stage classification process was deciding whether a project could be classified as a ‘public health research project’, for the reasons explained in the previous paragraphs. Therefore, the authors developed a quality assurance process to identify whether their developed inclusion and exclusion criteria could be easily applied to categorise projects as ‘public health research projects’. A second operator (without public health expertise) was given the inclusion and exclusion criteria for classifying projects as ‘public health research projects’ and asked to classify a sub-sample.

This inter-assessor agreement was assessed using Cohen’s kappa. This is a statistical measure of inter-operator agreement which is thought to be a more robust measure than percentage agreement, because it takes into consideration the likelihood of the agreement occurring by chance. A suggested sample size to distinguish between a kappa of 0.6 and 0.8 was calculated, for a power of 90 % and a significance level of 5 % which suggested 199 projects be assessed to calculate the kappa. This was calculated with the aid of the computer statistical package R® [17, 18].

### Procedure for sub-sample checking

Two hundred projects were selected at random with the help of the computer package Microsoft Excel®. The independent separate operator (without public health expertise) was provided the inclusion and exclusion criteria used in this study and was asked to judge whether the 200 projects could be classified as being related to public health research or not.

The second operator disagreed with the classification by the first operator in six cases. The Kappa was calculated as 0.94, with anything above 0.8 generally seen as indicating good reliability [19]. This suggested that the inclusion and exclusion criteria used for the study were specific enough to reliably include or exclude projects as being ‘public health research projects’. It is important to note that these criteria represent the views of the study’s authors and that universally recognised criteria do not exist.

### Coding according to the PHOF and the NICE taxonomy

The more difficult decision was whether a research project met the criteria for being classified as a ‘public health research project’. Once this decision had been made it was relatively simple to code the appropriate fields to the PHOF and the NICE taxonomy. Having one coder completing this task gave greater consistency for the vast amount of projects included in the study. However, any coding difficulties that presented were discussed with a colleague with public health expertise.

### Analysis with QlikView®

QlikView® is a business intelligence platform which enables straightforward analysis of complex data. It was used to prepare the charts and graphs in the following section.

## Results

The number and percentage of public health projects identified for each NIHR programme or school is shown below in Table 3.

**Table 3:** The number and percentage of public health research projects from the funding programmes and schools of the NIHR (2006-2013)

	No. of public health projects	Total No. of research projects	% of public health projects
Efficacy and Mechanism Evaluation (EME)	2	43	5
Health Services and Delivery Research (HS&DR)	186	305	61
Health Technology Assessment (HTA)	159	520	31
Health Technology Devices (HTD)	1	26	4
Invention for Innovation (i4i)	1	113	1
New and Emerging Applications of Technology (NEAT)	3	72	4
Programme Grants for Applied Research (PGfAR)	80	132	61
Programme Development Grant (PDG)	20	29	69
Public Health Research (PHR)	45	45	100
Research for Patient Benefit (RfPB)	164	457	36
Research for Innovation Speculation and Creativity (RISC)	2	17	12
School for Primary Care Research (SPCR)	44	89	49
School for Public Health Research (SPHR)	12	12	100
School for Social Care Research (SSCR)	41	54	76
Systematic Reviews (SR) Programme	17	67	25
Trainees’ Coordinating Centre (TCC)	176	1106	16
Total	953	3087	31

Some funding programmes had a low number of projects, for example EME and i4i, as their remit is related to basic science. Some programmes, such as SSCR, had a higher amount than expected, due to the programme funding various projects related to ‘improving the wider determinants of health’, for example relating to employment.

**Fig. 2:**
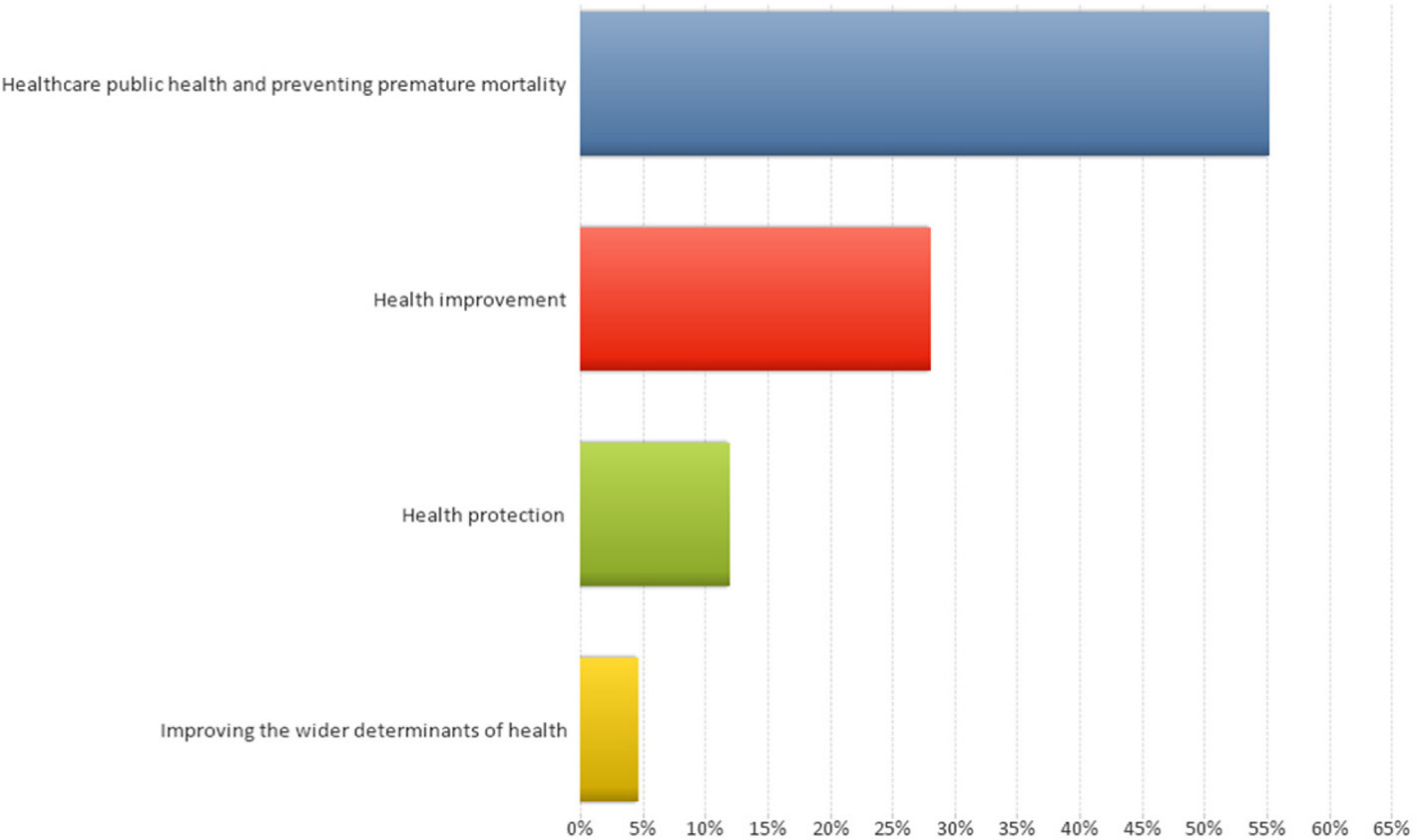
The percentage of public health research projects categorised according to the four domains of the Public Health Outcomes Framework

The most prevalent PHOF domain for the projects was ‘healthcare public health and preventing premature mortality’, which accounted for just over one-half of the projects. This category included both accessibility and redesign of healthcare services, and prevention of premature mortality. Only 5 % of projects were within the category of ‘improving the wider determinants of health’. The percentage of projects according to the four domains of the Public Health Outcomes Framework is shown in Fig. 2.

**Fig. 3:**
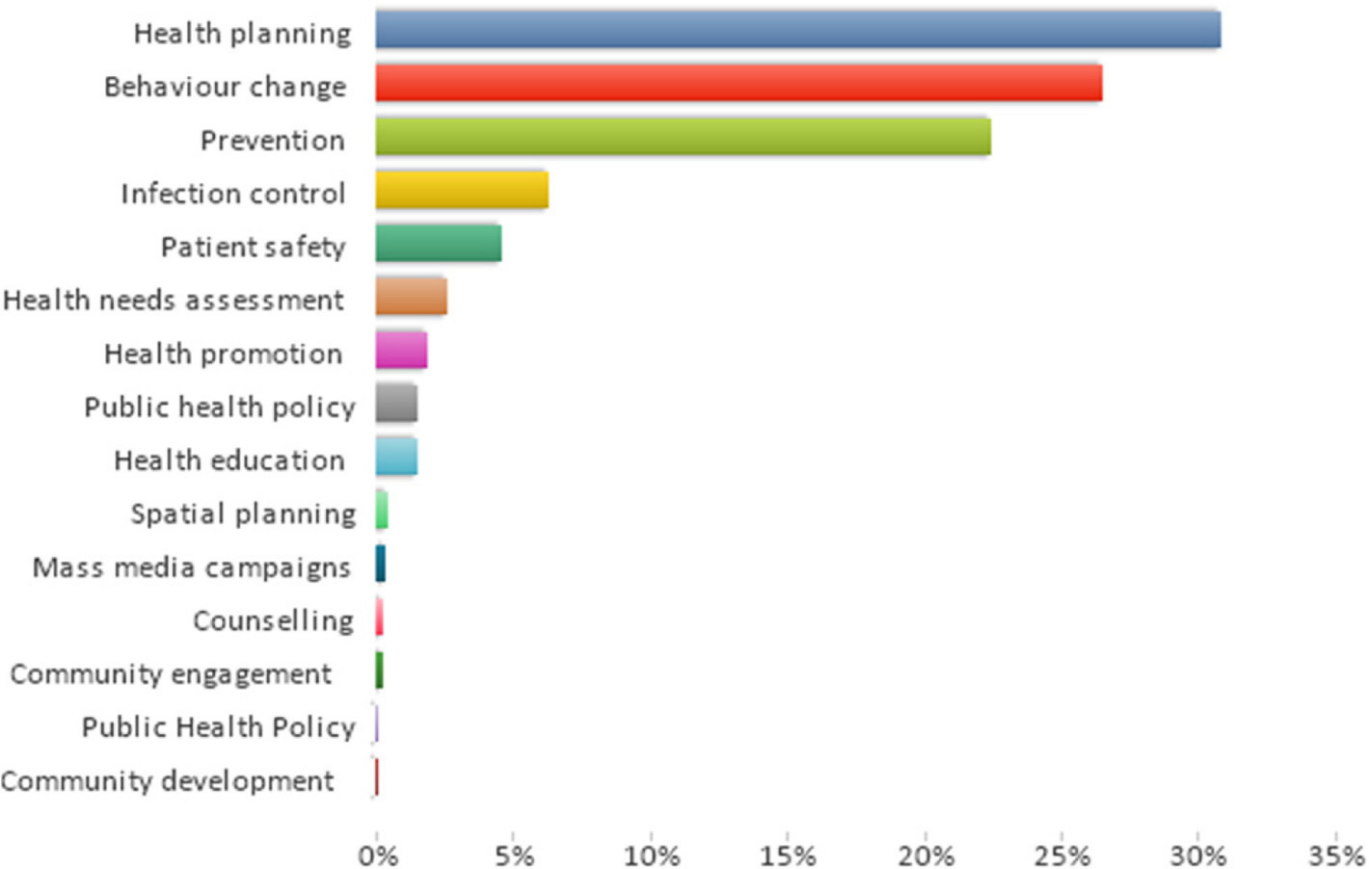
The percentage of public health research projects categorised according to ‘public health practice’, as defined by the NICE taxonomy

Just under one-third of projects concerned re-design and access to health services, which were categorised as being within ‘health planning’ according to the NICE taxonomy. The second most prevalent category was ‘behaviour change’, which accounted for approximately one-quarter of projects. The percentage of projects categorised according to ‘public health practice’ by the NICE taxonomy is shown below in Fig. 3.

**Fig. 4:**
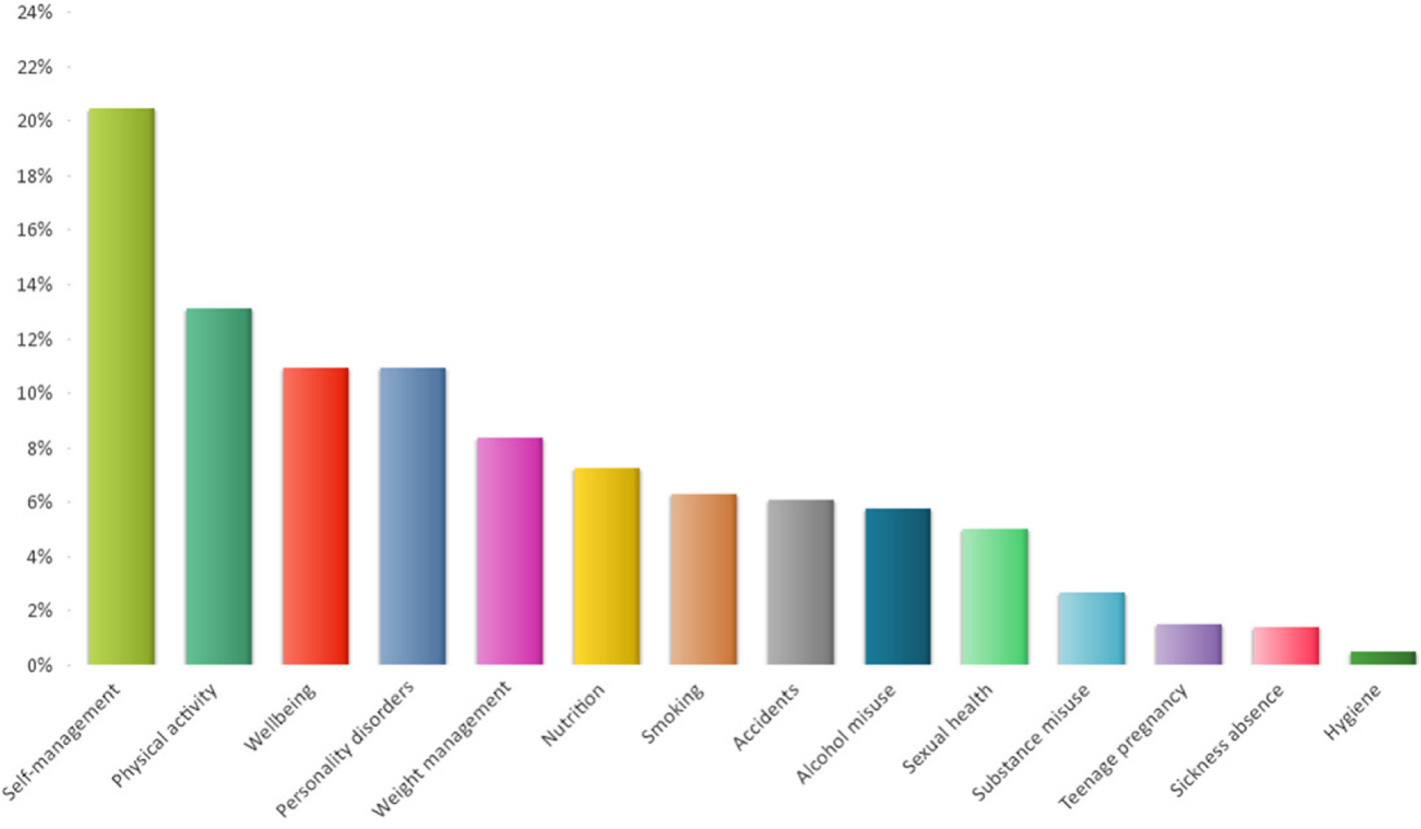
The percentage of public health research projects categorised according to ‘health behaviour’, as defined by the NICE taxonomy

The public health research projects which concerned behaviour change were further categorised with regard to the specific health behaviours covered and this is shown below in Fig. 4. The most prevalent category was ‘self-management’ of illnesses, such as asthma.

**Fig. 5:**
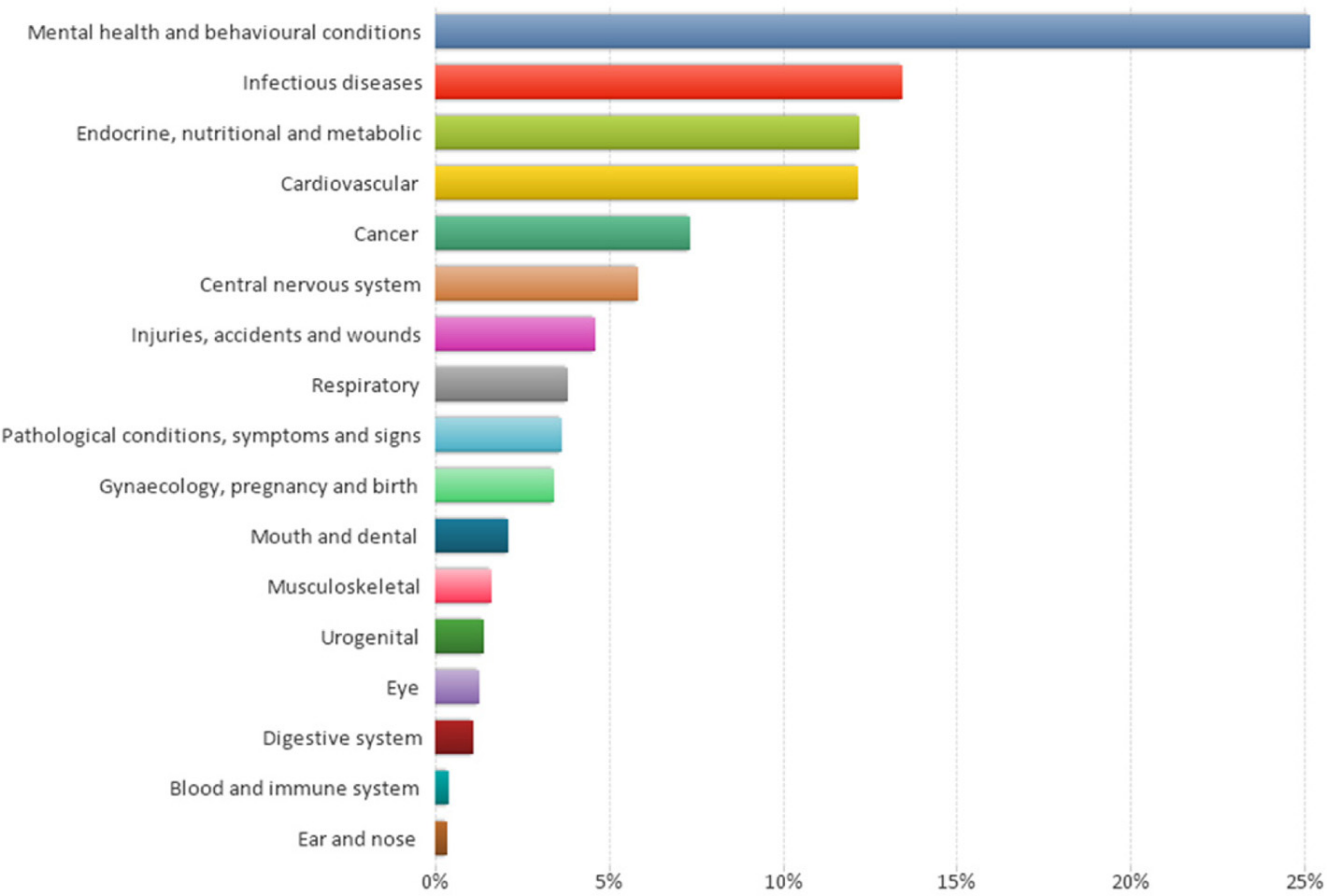
The percentage of public health research projects categorised according to illness or condition, as defined by the NICE taxonomy

The public health research projects were categorised into the illness/condition concerned (where appropriate) and this is displayed in Fig. 5.

The most prevalent category was mental health and behavioural conditions. This category includes both mental health conditions, such as depression and behavioural conditions, such as smoking.

## Discussion

This study reports on the breadth and balance of public health research funded by the UK NIHR over a 7 year period. Over one-third of its research portfolio could be classified as being related to public health, according to the inclusion and exclusion criteria used in this study. This reflects recognition of the importance of this type of research. Interestingly, this represents a greater proportion than that identified in a review of research funded by the National Institutes of Health in the United Stated of America in a 2 year period from 2010 to 2012. That portfolio analysis estimated that almost one-fifth of its total research expenditure was allocated to disease prevention, although this difference may reflect the classification systems used [20].

All of the NIHR research programmes and schools had funded at least one project classified as being relevant to public health, which reflects that public health research is funded across the breadth of the NIHR. Unsurprisingly, all of the research funded by the PHR Programme and the SPHR related to public health, as their remit specifies this type of research.

There was a continual growth of active public health research projects over time. This may be explained partly by the development of new programmes. For example, the NIHR’s core research is located within the NHS, with specific funding for public health research increasing during the study period. However, it also recognises the impact on the NHS, and on the health of the nation, of a broader range of interventions and settings. Given this broader view a gap was identified in the funding of high quality, nationally important, evaluations of public health interventions in non-NHS settings [21]. This lead to the start of the PHR Programme in 2008 and SPHR in 2012.

Research was funded across all four of the domains of the Public Health Outcomes Framework. The largest category was ‘healthcare public health and preventing premature mortality’, which included just over one-half of the public health research projects. Projects within ‘healthcare public health’ included those considering the accessibility and redesign of health services provided by the NHS. ‘Preventing premature mortality’ projects include those that aim to prevent mortality from conditions such as cardiovascular disease and diabetes. The large number of projects within the ‘healthcare public health and preventing premature mortality’ category reflects that the NIHR funds research on behalf of the NHS, social care and public health and puts patients and the public at the centre of everything they do. There were a very low number of projects funded within the category of ‘improving the wider determinants of health’. Projects funded within this area included those considering housing, transport and employment. In their 2010 study, Bambra et al. considered that modifying lifestyle interventions are often easier to identify and research than those concerning wider social determinants of health and health inequalities [22]. However, these types of projects may be covered by the PHR Programme and the SPHR, which are still in their early stages of funding studies. Furthermore, the DH Policy Research Programme (PRP), which supports research on the development and evaluation of ‘policy’, as distinct from ‘practice’, funds research on wider determinants of health, some of which is done in collaboration with other government departments.

The most prevalent illness/condition for the research projects was ‘mental health and behavioural conditions’, which accounted for approximately one-quarter of projects. As well as covering mood disorders, such as depression, this category also included self-harm, domestic violence and health behaviours, such as alcohol use, smoking and substance use. Mental health is a significant health concern in the UK, which has recently prompted a number of government strategies, such as ‘No health without mental health’ [23]. This emphasised the important link between good mental health, physical health, and wider social and economic benefits.

The second most prevalent illness or condition researched was ‘infectious diseases’ which accounted for 13 % of public health projects. These projects were primarily within the ‘health protection’ domain and, for example, included projects focused on vaccination programmes. The importance of this type of research has been highlighted to provide safe, cost effective and efficient means of preventing illness from infectious diseases. The high proportion of research projects echoes the value of this type of research, which was highly prevalent in the Wellcome Trust’s portfolio review [3]. Other ‘health protection’ studies are funded by NIHR or the PRP. However, the 13 NIHR Health Protection Research Units were not established until April 2013 and are therefore outside this study.

An important outcome of the study has been linking examples of NIHR funded public health research to public health guidelines issued by the National Institute for Health and Care Excellence (NICE). These are UK ‘guidelines on public health topics [that] make recommendations on local interventions that can help prevent disease or improve health’. Since February 2014 the guidelines have included a section for ‘Relevant ongoing NIHR research’. This gives public health professionals and academics examples of research studies related to the guideline topic that are in progress or at a pre-publication stage.

This study is limited because it is a descriptive analysis of the breadth and scope of public health research funded. To get an indication of the actual value of the research to practice would require future assessment of the impact of the research findings. This would be a useful exercise to reflect whether the NIHR is funding projects with direct relevance to improving population health. Measures of impact can include citation in scientific publications, citation in public health guidance and inclusion in information relevant to policy. However, for many research projects in this portfolio analysis it is considered too early to assess their impact at this time. This is an important consideration for the future. Approaches to assess impact could include analysis of case studies and qualitative interviews of study investigators and evidence users.

The main limitation of this study was defining what exactly constitutes a ‘public health research project’ and therefore which projects should be included in the study. Obtaining views from specialists within the public health field revealed many different opinions of what should be included, relating to the wide range of practice and interests within public health. The inclusion criteria were purposively wide, to capture projects within all four of the domains of the Public Health Outcomes Framework. A similar difficulty in classifying public health research projects was experienced by researchers who compiled an inventory of Swedish public health research in 2005 [8]. The authors of a report of public health research funded in Europe also emphasised the difficulties in agreeing on a concept of public health [24]. An overview of the literature for European research in health management over a 10 year period from 1995 to 2005 concluded that, ‘Public health is not a well-defined discipline with clear boundaries in research terms since it includes contributions from a wide range of social and behavioural sciences’ [25]. Consequently, this has made the task of searching the databases both especially complicated and perhaps a more subjective exercise than might have been desirable.

Another major limitation for this study’s methodology is that a universally recognised taxonomy for public health research projects does not exist. Therefore, using alternative taxonomies may present a different picture of the research. The NICE taxonomy was used for this study and enabled linking examples of NIHR funded public health research projects to NICE public health guidelines. The Public Health Outcomes Framework was also used because this was developed by the Department of Health in 2012 as a way of measuring outcomes for the public health system [13], and so it applies to UK public health practice. These coding systems were deemed relevant and acceptable for use in the current study. However, the classification schemes used, and other possibilities identified, all have limitations that can affect generalisability to other settings. The lack of a universally accepted classification scheme for public health research is a barrier to the reproducibility and comparability of results. Therefore the authors suggest that developing a public health-specific taxonomy is a priority for the public health community. The need for the development of a better classification scheme is an identified knowledge gap from this work. In this context, it is interesting to note that a classification of public health interventions is being developed for the World Health Organization International Classification of Health Interventions. The classification aims to provide ‘a common tool for reporting and analysing the distribution and evolution of health interventions for statistical purposes’ [26]. The inclusion of public health interventions is a development of interest in this field.

Mapping a research funder’s public health portfolio enables identification of the number and type of research projects within specific areas, such as by illnesses and conditions. This can pave the way for the identification of priority areas for the commissioning of future research. Appropriate research priority-setting is an integral part of a needs-led research agenda. Future evaluation is recommended to ensure the value of research, for example continuing to evaluate the portfolio by further in-depth analysis in particular areas, such as health inequalities or specific population groups.

## Conclusions

Approximately one-third of the UK NIHR’s research portfolio (2006–2013) was classified as being relevant to public health, according to the inclusion and exclusion criteria used in this study. This analysis offers a snapshot of the breadth and balance of NIHR research, which forms a basis for discussion and to identify areas for possible future research commissioning. Although this research has a number of limitations it provides an opportunity to reflect on the portfolio of public health research funded by one of the foremost funders of health research in the UK. Future research could consider applying other taxonomies or classifications of public health interventions, and assessing the value and impact of the public health research that has been funded.

## Additional files

Additional file 1: Classification as Public Health Research. (DOCX 17 kb) Additional file 2: NICE taxonomy codes. (DOCX 24 kb)

## Abbreviations

HRCS: Health Research Classification System
NIHR: National Institute for Health Research
PHIRE: Public Health Innovation and Research in Europe
PHOF: Public Health Outcomes Framework
SPHERE: Strengthening Public Health Research in Europe
UKCRC: UK Clinical Research Collaboration

## References

[1] Chalmers I, Bracken MB, Djulbegovic B, Garattini S, Grant J, Gülmezoglu AM (2014). How to increase value and reduce waste when research priorities are set. Lancet.

[2] UK Clinical Research Collaboration. UK Health Research Analysis 2009/10. London UK Clinical Research Collaboration 2012

[3] Wellcome Trust (2013). Portfolio Review; Population and Public Health 1990–2011.

[4] European Commission (2012). SPHERE: Strengthening Public Health Research in Europe.

[5] McCarthy M, Clarke A (2007). European public health research literatures—measuring progress. Eur J Public Health.

[6] McCarthy M, Dyakova M, Clarke A (2014). Public health research in the UK: a report with a European perspective. J Public Health.

[7] McCarthy M, Alexanderson K, Conceicao C, Barnhoorn F, Grimaud O, Katreniakova Z, et al. PHIRE - Public Health Innovation and Research in Europe: Summary Report. The Netherlands: European Public Health Association; 2013.

[8] Kallestal C, Swanberg I (2005). Part 2: An inventory of Swedish public health research. Scand J Public Health.

[9] Gulis G, Eriksen ML, Aro AR (2010). Public health research in Denmark in the years 1995–2005. Scand J Public Health.

[10] National Institute for Health Research. 2016. http://www.nihr.ac.uk. Accessed 18 Mar 2016.

[11] Department of Health (2006). Best Research for Best Health.

[12] National Institute for Health Research. National Institute for Health Research: Vision, Mission, Aims. 2016. http://www.nihr.ac.uk/about/mission-of-the-nihr.htm. Accessed 18 Mar 2016.

[13] Department of Health (2012). The Public Health Outcomes Framework for England, 2013–2016.

[14] U.S. National Library of Medicine. Medical Subject Headings (MeSH®). 2015. https://www.nlm.nih.gov/pubs/factsheets/mesh.html. Accessed 18 Mar 2016.

[15] National Public Health Language. 2007. http://www.apho.org.uk/resource/view.aspx?RID=39629. Accessed 18 Mar 2016.

[16] National Institutes for Health and Care Excellence. NICE Taxonomy. 2012. https://www.nice.org.uk. Accessed Mar 2016.

[17] kappaSize: Sample Size Estimation Functions for Studies of Interobserver Agreement. https://cran.r-project.org/web/packages/kappaSize/kappaSize.pdf. Accessed 18 Mar 2016.

[18] R Foundation for Statistical Computing. Statistical Package R: R: A language and environment for statistical computing. Vienna: http://www.R-project.org; 2009.

[19] Carletta J (1996). Assessing agreement on classification tasks: the kappa statistic. Computational linguistics.

[20] Calitz C, Pollack K, Millard C, Yach D (2015). National Institutes of Health Funding for behavioral interventions to prevent chronic diseases. Am J Prev Med.

[21] Milne R, Law C (2009). The NIHR public health research programme: developing evidence for public health decision-makers. J Public Health.

[22] Bambra C, Gibson M, Sowden A, Wright K, Whitehead M, Petticrew M (2010). Tackling the wider social determinants of health and health inequalities: evidence from systematic reviews. J Epidemiol Community Health.

[23] HM Government, Department of Health. No health without mental health: A cross-government mental health outcomes strategy for people of all ages. London: Crown copyright; 2011.

[24] Conceição C, Leandro A, McCarthy M (2009). National support to public health research: a survey of European ministries. BMC Public Health.

[25] Hunter D, Brown J (2007). A review of health management research. Eur J Public Health.

[26] World Health Organization. International Classification of Health Interventions (ICHI). 2016. http://www.who.int/classifications/ichi/en. Accessed 18 Mar 2016.

