# Supplementary material for: Mapping public health research across the National Institute for Health Research 2006–2013
Source: BMC Public Health. 2016 Aug 31;16(1):911. doi: 10.1186/s12889-016-3521-z (PMC5007681; doi:10.1186/s12889-016-3521-z)
Supplement: Additional file 1: — Classification as Public Health Research. (DOCX 17 kb) [file 12889_2016_3521_MOESM1_ESM.docx]

**Additional file 1: Classification as Public Health Research**

Colleagues, including those with a public health background, had varying ideas about what constituted a ‘public health research project’. Therefore it was necessary to derive Inclusion and Exclusion criteria.

**Included Studies**

• Preventative interventions and health promotion;

Interventions, including treatments, to prevent illnesses/conditions e.g. stopping smoking, sexual health, physical activity, breastfeeding, community groups and engagement, self-harm and suicide prevention, alcohol, teenage pregnancy, health promotion, substance misuse, domestic violence, suicide prevention, alerts for those living alone, obesity, teenage pregnancy, prevention of home and traffic accidents, employment, offender health, victims of crime

• Health education, health literacy

• Early clinical diagnosis/identification (but excluding those that concern development of diagnostic technologies)

• Screening programmes; design of programmes, improvement of programmes

• Identification of clinical thresholds and care pathways for common conditions

• Perinatal and postnatal mental disorders – both identification and treatment of these conditions (due to effect on foetuses/babies)

• Health inequalities

• Accessibility of care

• Self-management and self-care behaviours; self-monitoring, education, self-help

• Improving services, investigations about new models of service provision, exploring variations in hospitals’ services, primary care, secondary care, social care, pharmacy, ambulance etc. Including patient involvement, health needs assessment and health planning, care pathways, reducing unscheduled care, variations in morbidity/mortality rates, equity of access, accessibility of healthcare, provision of care at home instead of in hospital, professional communities and networks, location of end-of-life care, telemonitoring, e-HEALTH, planning for long-term needs for conditions, discharge planning, integrated care & interface management, alternative provision, governance

• Socioeconomic problems – crime, housing, environment etc.

• Health protection;

Patient safety; safety of health services, adverse drug reactions, safety of patient care, infection control, prevention of spread of infectious diseases, vaccinations to prevent infectious diseases, uptake of vaccination programmes by patients, mass media campaigns, reduction in antimicrobial resistance, prevention of spread of infectious diseases including tuberculosis, HIV, threat detection, infection surveillance.

**Excluded Studies**

• Secondary preventative – e.g. reducing the risk of relapse of depression etc.

• Basic science and clinical studies, biobanks, registries

• Treatment (fertility, rehabilitation, physiotherapy, medicines, radiotherapy, CBT, or surgical interventions for pre-existing illness/conditions (with the exception of treatments for perinatal mental disorders)

• Development of diagnostic tests, treatment devices, decontamination of equipment

• Workforce issues of leadership and training issues, development of measures to enable evaluation of patient experience, culture changes for organisations, roles of staff groups, peer support

• Development of data collection tools, identification of research priorities, development of outcome measures, recruitment to trials

• Descriptive epidemiological studies

• Healthcare management studies – team working, leadership, training issues.
