# Supplementary material for: Mapping public health research across the National Institute for Health Research 2006–2013
Source: BMC Public Health. 2016 Aug 31;16(1):911. doi: 10.1186/s12889-016-3521-z (PMC5007681; doi:10.1186/s12889-016-3521-z)
Supplement: Additional file 2: — NICE taxonomy codes. (DOCX 24 kb) [file 12889_2016_3521_MOESM2_ESM.docx]

**Additional file 2: Public Health practice**

**NICE codes used for the Public Health practice categories**

In some cases there could be more than one Public Health practice – therefore there were two category fields for this field, both of one level.

| **Level 1** | **Level 2** |
| --- | --- |
| Behaviour change | - |
| Community development | - |
| Community engagement | - |
| Counselling | - |
| Health education | - |
| Health needs assessment | - |
| Health planning | - |
| Health promotion | - |
| Infection control | - |
| Mass media campaigns | - |
| Patient safety | - |
| Prevention | - |
| Public health policy | - |
| Spatial planning | - |

**NICE codes used for the Health Behaviour categories**

In some cases there could be more than one Health Behaviour – therefore there were two category fields for this field, both of two levels.

| **Level 1** | **Level 2** |
| --- | --- |
| Accidents | Home accidents  Traffic accidents |
| Alcohol misuse |  |
| Hygiene |  |
| Nutrition | Breastfeeding  Healthy eating |
| Personality disorders | Disruptive and violent behaviour  Self-harm |
| Physical activity |  |
| Self-management |  |
| Sexual health |  |
| Sickness absence |  |
| Smoking |  |
| Substance misuse | Drug misuse |
| Teenage pregnancy |  |
| Weight management |  |
| Wellbeing |  |

**NICE codes used for the Treatments, procedures and devices categories**

In some cases there could be more than one Treatments, procedures and devices – therefore there were two category fields for this field, both of three levels.

| **Level 1** | **Level 2** | **Level 3** |
| --- | --- | --- |
| Clinical equipment and devices | Catheters  Diagnostic devices  Surgical devices |  |
| Diagnostic, therapeutical and surgical procedures | Care pathway  Diagnostic procedures  Surgical procedures  Therapeutic procedures | Screening  Blood pressure monitoring  Radiology  Bones and joint surgery  Heart, arteries and veins surgery  Miscellaneous surgical procedures  Transplantation  Cognitive behavioural therapy  Counselling  Dialysis  Rehabilitation |
| Drug treatments | Cardiovascular system (drug treatment)  Central nervous system (drug treatments)  Endocrine system (drug treatments)  Immunological products and vaccines  Infections (drug treatments)  Musculoskeletal and joint (drug treatments) |  |
| Patient care |  |  |

**NICE codes used for the Socioeconomic determinants of health category**

| **Level 1** | **Level 2** |
| --- | --- |
| Accessibility | - |
| Culture | - |
| Employment | - |
| Environment | - |
| Ethnicity | - |
| Homelessness | - |
| Housing | - |
| Income | - |
| Inequalities | - |
| Lifestyle | - |
| Social class | - |
| Social exclusion | - |

**NICE codes used for the Target population categories**

In some cases there could be more than one Target Population – therefore there were two category fields for this field, both of three levels.

| **Level 1** | **Level 2** | **Level 3** |
| --- | --- | --- |
| Age groups | Adults  Children | Young people  Older people  Fetuses  Infants  Neonates  Pre school children |
| Disabled people | People with a learning disability  People with a physical disability | - |
| Employment group | Employees  [Healthcare professionals] | - |
| Ethnic groups | Asian people  Black people | - |
| Gender | Female  Male | - |
| Patients and carers | Carers  Inpatients  Outpatients | - |
| Relationships | Families | Parents  Partners |
| Sexuality | Homosexual | - |
| Vulnerable groups | Homeless people  Looked after children  Low income people  Migrants  Offenders  Sex workers  Substance misusers  Travellers | - |

*Note; Healthcare professionals do not fit coding structure – but classified as employment group*

**NICE codes used for the Illness or condition categories**

In some cases there could be more than one Illness or condition – therefore there were two category fields for this field, both of three levels.

| **Level 1** | **Level 2** | **Level 3** |
| --- | --- | --- |
| Blood and immune system | Sickle cell anaemia |  |
| Cancer | Breast cancer  Cervical cancer  Gastrointestinal cancer  Endometrial cancer  Head and neck cancer  Lung cancer  Lymphoma  Myeloma  Oesophageal cancer  Oral cancer  Skin cancer  Urogenital cancer | Bowel cancer  Colorectal cancer  Bladder cancer  Prostate cancer |
| Cardiovascular | Vascular diseases  Heart diseases | Coronary disease  Embolism  Hypertension  Peripheral arterial disease  Stroke  Acute coronary syndrome  Angina  Heart failure  Myocardial infarction |
| Central nervous system | Cerebral palsy  Chronic fatigue syndrome  Creutzfeldt jakob disease  Dementia  Epilepsy  Headache  Motor neurone disease  [pain]  Parkinson disease |  |
| Digestive system | Gastrointestinal diseases  Liver diseases | Colitis  Crohn’s disease  Hernia  Liver cirrhosis |
| Disabilities |  |  |
| Ear and nose | Hearing disorders |  |
| Endocrine, nutritional and metabolic | Diabetes  Obesity  Thyroid diseases  Vitamin deficiency | Type I diabetes  Type II diabetes  Hypothyroidism  Hyperthyroidism |
| Eye | Glaucoma  Macular degeneration  Vision disorders |  |
| Gynaecology, pregnancy and birth | Birth  Gynaecology  Pregnancy | Endometriosis |
| Infectious diseases | Bacterial infections  Viral infections  Sexually transmitted infections | Methicillin resistant S.aureus  Hepatitis  Influenza  Chlaymdia  HIV infection |
| Injuries, accidents and wounds | Burns  Fractures  Head injury |  |
| Mental health and behavioural conditions | Autism spectrum disorders  Eating disorders  Learning disabilities  Mood disorders  Perinatal mental disorders  Personality disorders  Psychosis  Schizophrenia  Substance-related disorder | Autism  Asperger syndrome  Bipolar disorders  Depression  Anxiety  Self-harm  Violent and disruptive behaviour  Alcohol misuse  Smoking  Substance misuse |
| Mouth and dental | - |  |
| Musculoskeletal | Bone disorders | Arthritis  Osteoarthritis |
| Non-communicable diseases | - |  |
| Pathological conditions, symptoms and signs | Pathological processes  Symptoms and signs | Disease attributes  Neurological symptoms |
| Respiratory | Asthma  Bronchitis  Chronic obstructive pulmonary disease  Cystic fibrosis  Dyspnoea  Tuberculosis |  |
| Skin | Eczema |  |
| Urogenital | Chronic kidney disease  Renal failure  Urinary tract infection |  |
| *Conditions not listed in the NICE taxonomy* | *[Congenital abnormality]*  *[Dehydration]*  *[Dizziness]*  *[Dysphagia]*  *[Genetic disorder]*  *[Hypotension]* |  |

**NICE codes used for the Settings categories**

In some cases there could be more than one Setting – therefore there were two category fields for this field, both of three levels.

| **Level 1** | **Level 2** | **Level 3** |
| --- | --- | --- |
| Community settings | Care settings | Adult social care  Child social care  Intermediate care homes  Residential care homes  Residential child care |
|  | Education settings | Further and higher education  Nursery schools  Primary schools  Secondary schools |
|  | Home |  |
|  | Places of worship |  |
|  | Prisons |  |
|  | Recreation settings | Sport and leisure facilities |
|  | Rural areas |  |
|  | Temporary accommodation |  |
|  | Transport infrastructure |  |
|  | Urban areas |  |
|  | Workplaces |  |
|  |  |  |
| Healthcare settings | Ambulances |  |
|  | Clinics |  |
|  | General practice |  |
|  | Hospices |  |
|  | Hospitals | Accident and emergency wards  Critical care wards  General wards  Mental health wards  Operating departments |
|  | Pharmacies |  |
